# Two-stage genome-wide association study identifies a novel susceptibility locus associated with melanoma

**DOI:** 10.18632/oncotarget.15230

**Published:** 2017-02-09

**Authors:** Katherine J. Ransohoff, Wenting Wu, Hyunje G. Cho, Harvind C. Chahal, Yuan Lin, Hong-Ji Dai, Christopher I. Amos, Jeffrey E. Lee, Jean Y. Tang, David A. Hinds, Jiali Han, Qingyi Wei, Kavita Y. Sarin

**Affiliations:** ^1^ Department of Dermatology, Stanford University School of Medicine, Stanford, CA, USA; ^2^ Department of Epidemiology, Richard M. Fairbanks School of Public Health, Melvin and Bren Simon Cancer Center, Indiana University, Indianapolis, IN, USA; ^3^ Department of Epidemiology and Biostatistics, Tianjin Medical University Cancer Hospital and Institute, National Clinical Research Center for Cancer, Tianjin and Key Laboratory of Cancer Prevention and Therapy, Tianjin, China; ^4^ Department of Community and Family Medicine, Geisel School of Medicine, Dartmouth College, Hanover, NH, USA; ^5^ Department of Surgical Oncology, The University of Texas M.D. Anderson Cancer Center, Houston, TX, USA; ^6^ 23andMe Inc., Mountain View, CA, USA; ^7^ Department of Epidemiology, Harvard T.H. Chan School of Public Health, Boston, MA, USA; ^8^ Duke Cancer Institute, Department of Medicine, Duke University, Durham, NC, USA

**Keywords:** melanoma, genome-wide association study, single nucleotide polymorphism, susceptibility loci, BASP1

## Abstract

Genome-wide association studies have identified 21 susceptibility loci associated with melanoma. These loci implicate genes affecting pigmentation, nevus count, telomere maintenance, and DNA repair in melanoma risk. Here, we report the results of a two-stage genome-wide association study of melanoma. The stage 1 discovery phase consisted of 4,842 self-reported melanoma cases and 286,565 controls of European ancestry from the 23andMe research cohort and the stage 2 replication phase consisted of 1,804 melanoma cases and 1,026 controls from the University of Texas M.D. Anderson Cancer Center. We performed a combined meta-analysis totaling 6,628 melanoma cases and 287,591 controls. Our study replicates 20 of 21 previously known melanoma-loci and confirms the association of the telomerase reverse transcriptase, *TERT*, with melanoma susceptibility at genome-wide significance. In addition, we uncover a novel polymorphism, rs187843643 (OR = 1.96; 95% CI = [1.54, 2.48]; *P* = 3.53 × 10^−8^), associated with melanoma. The SNP rs187842643 lies within a noncoding RNA 177kb downstream of *BASP1* (brain associated protein-1). We find that *BASP1* expression is suppressed in melanoma as compared with benign nevi, providing additional evidence for a putative role in melanoma pathogenesis.

## INTRODUCTION

Melanoma represents 1% of cutaneous malignancies, affecting approximately 76,000 people per year in the U.S, but causes the majority of skin cancer deaths [1]. Environmental risk factors such as ultraviolet radiation and fair skin pigmentation contribute to melanoma development [2]. About 10–12% of melanomas occur in familial clusters; to date, approximately half of the genes responsible for these clusters have been identified [3]. Prior genome-wide association studies (GWAS) have identified 21 loci associated with cutaneous melanoma (Supplementary Table 2). These previous studies implicate nevus count, pigmentation, telomere homeostasis, tumor suppression and DNA repair in melanoma development.

Crowd-sourced data has recently been utilized to identify susceptibility loci for a wide range of disease phenotypes [4]. Here, we utilize crowd-sourced data in a two-stage genome-wide association meta-analysis for melanoma, totaling 6,628 cases and 287,591 controls. In this GWAS, we replicate 20 of 21 previously identified melanoma-associated loci and discover one novel susceptibility locus at genome-wide significance.

## RESULTS

**Figure 1:**
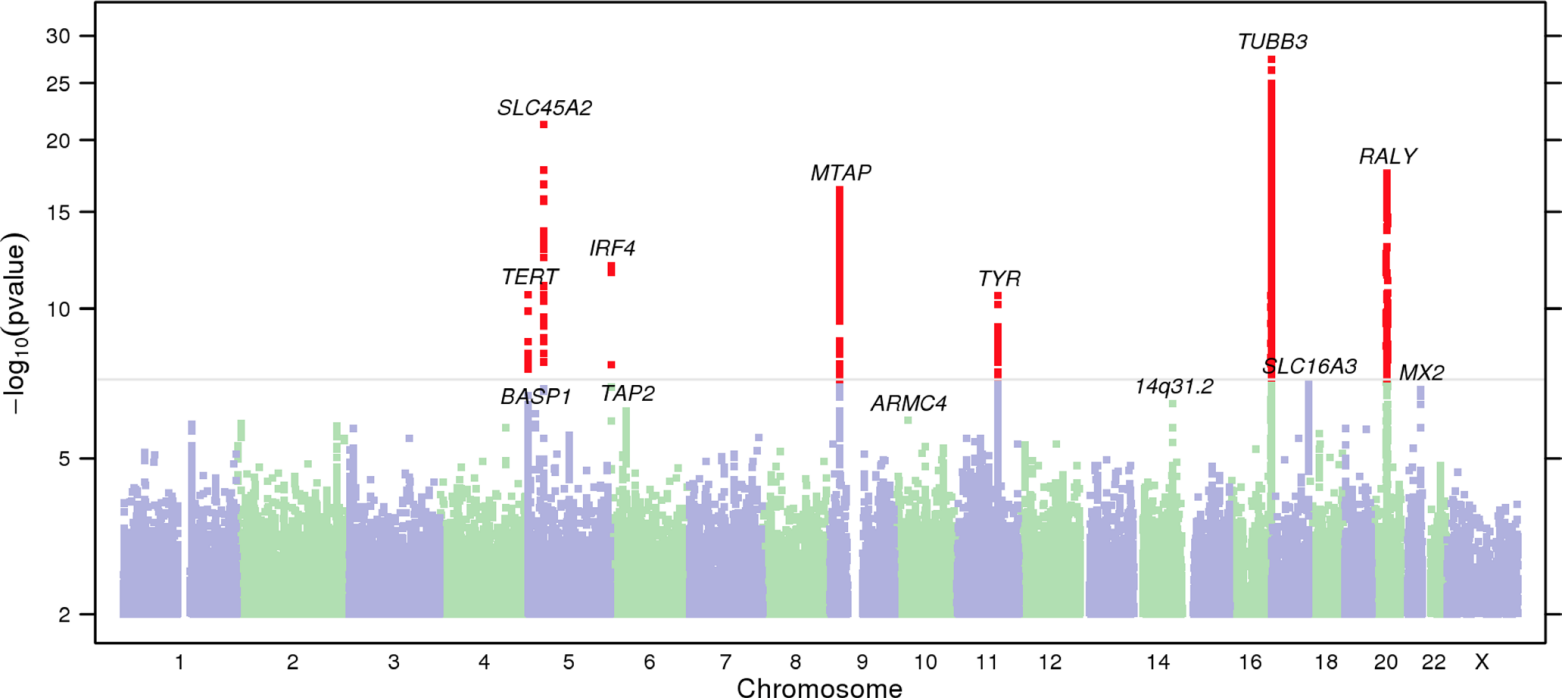
Manhattan plot of stage 1 GWAS analysis of melanoma from 23andMe dataset Total stage 1 GWAS analysis included 4,842 melanoma cases and 286,565 controls. SNPs with *P* < 5 × 10^−8^ are shown in red. Loci with smallest *P* < 10^−6^ are labeled with the name of the nearest gene. In stage 1, 13 loci reached genome-wide significance after adjusting for genomic control. One novel locus, 5p15.1 (*BASP1*—[]) was genome-wide significant in the overall meta-analysis (Table 1).

Stage 1 consisted of 4,842 self-reported melanoma cases and 286,565 controls of European ancestry from the 23andMe, Inc. research cohort (Table 1). A validation of self-report of melanoma history using the same 23andMe survey questions with adjudicated medical records revealed a sensitivity of 100% and specificity of 98.8% (*p* < 0.0001; Fisher's exact test) (Supplementary Table 1). The most significant melanoma-associated SNP at each locus (*P* < 10^−5^) was identified, resulting in nine index single nucleotide polymorphisms (SNPs) (Table 2, Figure 1). Stage 2 consisted of 1,804 melanoma cases and 1,026 non-hispanic controls from the MD Anderson Cancer Center. Four of the nine index SNPS were replicated in the stage 2 analysis (*P* < 0.05). Although some loci did not reach statistical significance in stage 2, their 95% confidence intervals (for odds ratios) overlapped with the corresponding stage 1 confidence intervals.

**Table 1:** Gender and age of melanoma cases and controls from GWAS

	Status	*n* (%)	Male (%)	Age ≤ 30 yr	Age 30–45	Age 45–60	Age > 60
**23andMe (Stage 1) (*n*** **= 291,389)**	*Cases*	4,824 (1.66)	2587 (53.6)	99 (2)	476 (9.9)	1,203 (24.9)	3,056 (63.3)
	*Controls*	286,565 (98.3)	154,517 (53.9)	40,028 (13.9)	8,4188 (29.3)	7,8037 (27.2)	8,4312 (29.4)
**MD Anderson (Stage 2) (*n*** **= 2,830)**	*Cases*	1,804 (63.7)	1,060 (58.8)	142 (7.9)	441 (24.5)	684 (37.9)	536 (29.7)
	*Controls*	1,026 (36.3)	613 (59.8)	69 (6.7)	231 (22.5)	486 (47.4)	240 (23.4)
**Combined analysis (*n*** **= 294,219)**	*Cases*	6,628 (2.25)	3677 (2.3)	241 (3.6)	9,171 (13.8)	1,887 (28.4)	3,592 (54.2)
	*Controls*	287,591 (97.7)	155,130 (97.7)	40,097 (13.9)	84,419 (29.3)	78,523 (27.3)	84,552 (29.4)

Counts and percentages for cases and controls (*n* (%)) are listed above, stratified by stage of GWAS. We also report number and percentage of male subjects, subjects with age < 30 years, subjects with age 30–45 years, subjects with age 45–60 years, and subjects with age > 60 years.

**Table 2:** Loci reaching genome-wide significance in melanoma GWAS

SNP	Region	Gene	Maj/Min	Avg. imputation r^2^	MAF^1^	23&Me (Stage 1)		MD Anderson (Stage 2)		Meta-Analysis^2^	
						P	OR [CI^3^]	P	OR [CI^3^]	P	OR [CI^3^]
rs1805007	16q24.3	MC1R	C/T	1.00	0.07	3.8 × 10^−28^	1.47 [1.38, 1.57]	4.88 × 10^-8^	1.72 [1.41,2.09,]	4.24 × 10^-37^	1.50 [1.41,1.59]
rs35407	5p13.2	SLC45A2	G/A	0.98	0.03	5.6 × 10^−22^	0.46 [0.39, 0.55]	6.44 × 10^-3^	0.57 [0.38,0.85]	3.42 × 10^-19^	0.48 [0.41,0.56]
rs6059655	20q11.22	RALY-ASIP	G/A	0.99	0.07	3.1 × 10^−18^	1.37 [1.28, 1.46]	1.02 × 10^-4^	1.44 [1.20,1.74]	5.04 × 10^-23^	1.38 [1.29,1.47]
rs201131773	9p21.3	MTAP	I/D	0.99	0.48	2.4 × 10^−17^	1.19 [1.15, 1.24]	-	-	2.5 × 10^−17^	1.19 [1.15,1.24]
rs62389423	6p25.3	IRF4-[]--EXOC2	G/A	0.78	0.14	9.7 × 10^−13^	1.26 [1.18, 1.34]	7.77 × 10^-2^	1.15 [0.98,,1.34]	9.14 × 10^-14^	1.24 [1.17,1.31]
rs139996880	5p15.33	TERT	G/A	0.65	0.16	2.4 × 10^−11^	1.25 [1.18, 1.34]	0.48*	1.06* [0.90,1.26]	7.16 × 10^-12^	1.26 [1.18,1.34]
rs1393350	11q14.3	TYR	G/A	1.00	0.27	2.7 × 10^−11^	1.17 [1.11, 1.22]	8.47 × 10^-3^	1.18 [1.04,1.33]	3.65 × 10^-13^	1.17 [1.12,1.22]
rs45430	21q22.3	MX2	T/C	1.00	0.34	1.1 × 10^−7^	0.89 [0.85, 0.93]	0.11	0.91 [0.82,1.02]	2.89 × 10^-8^	0.89 [0.86,0.93]
rs187843643	5p15.1	BASP1---[]**	C/T	0.74	0.01	6.0 × 10^−7^	1.96 [1.54,2.50]	0.52	1.69 [0.34,8.38]	3.53 × 10^-8^	1.96 [1.54,2.48]

SNPs that met genome-wide significance (*P* < 5 × 10^−8^) in the overall meta-analysis are listed. Additionally, we report genetic locus, nearest genes, major allele, minor allele, minor allele frequency (MAF) in stage 1 controls, average imputation r^2^ (a measure of imputation quality) for stage 1, and odds ratio (OR) with *P* value for each stage, calculated with respect to the minor allele. Stage 1 included 4,842 melanoma cases and 286,565 controls from 23andMe. Stage 2 included 1,804 melanoma cases and 1,026 controls from the MD Anderson Cancer Center. The combined fixed-effect meta-analysis, totaled 6,628 melanoma cases and 287,591 controls. Statistics for effect heterogeneity (*P*_het_ and *I^2^*) are included in Supplementary Table 4. All subjects were from the US and of European ancestry.

^1^MAF = minor allele frequency in stage 1 controls

^2^Meta-analysis = Combined 23&Me + MD Anderson

^3^CI = 95% confidence interval

** = Not previously associated with melanoma risk

* = Imputation r^2^ = 0.2968 in MD Anderson Dataset.

A combined meta-analysis of stage 1 and stage 2, totaling 6,628 melanoma cases and 287,591 controls identified nine susceptibility loci reaching genome-wide significance (Table 2; *P* < 5 × 10^−8^). Out of 21 previously known melanoma-associated loci, 20 were replicated at nominal *p value* (*P* < 0.05, logistic regression) (Supplementary Table 2). We identified one novel melanoma-susceptibility locus, located at 5p15.1 (rs187843643; *BASP1—[]*) (P = 3.53 × 10^−8^; OR = 1.96, logistic regression). Further information on methods and imputation quality control can be found in Supplementary Methods, and Supplementary Tables 3, 4. QQ plots, forest plots, and regional association plots are provided in Supplementary Figures 1–3.

To measure the heritability of melanoma attributable to the genome-wide significant SNPs from stage 1, we calculated the familial relative risk for melanoma outlined by the Cancer Oncological Gene Environment Study (COGS) [5]. The nine loci explained 9% of familial melanoma risk. The novel SNP rs187843643 contributes to 1% of familial melanoma risk.

## DISCUSSION

Of the nine genome-wide significant SNPs resulting from the combined meta-analysis, eight were at previously identified loci associated with melanoma: *MC1R, SLC45A2, RALY, MTAP, IRF4-EXOC2, TERT, TYR*, and *MX2*. These loci are associated with pigmentation phenotype and nevus count. The pigmentation loci include 5p13.2 (*SLC45A2*), 11q14.3 (*TYR*), 16q24.3 (*MC1R*) and 20q11.22 (*RALY/ASIP*); nevi-associated loci are 9p21.3 (*CDKN2A-MTAP*), 22q13.1 (*PLA2G6*). The SNP rs6059655, intergenic near *RALY-ASIP*, is associated with facial pigmentation spots [6] and rs35407 (*SLC45A2)*, is associated with pigmentation and melanoma risk [7, 8]. It is important to note that some loci associated with pigmentation phenotype may also contribute to melanoma risk independent of pigmentation. For example, in addition to affecting hair color, previous studies have demonstrated that primary human melanocytes with *MC1R* variants have impaired DNA-repair [9].

**Figure 2:**
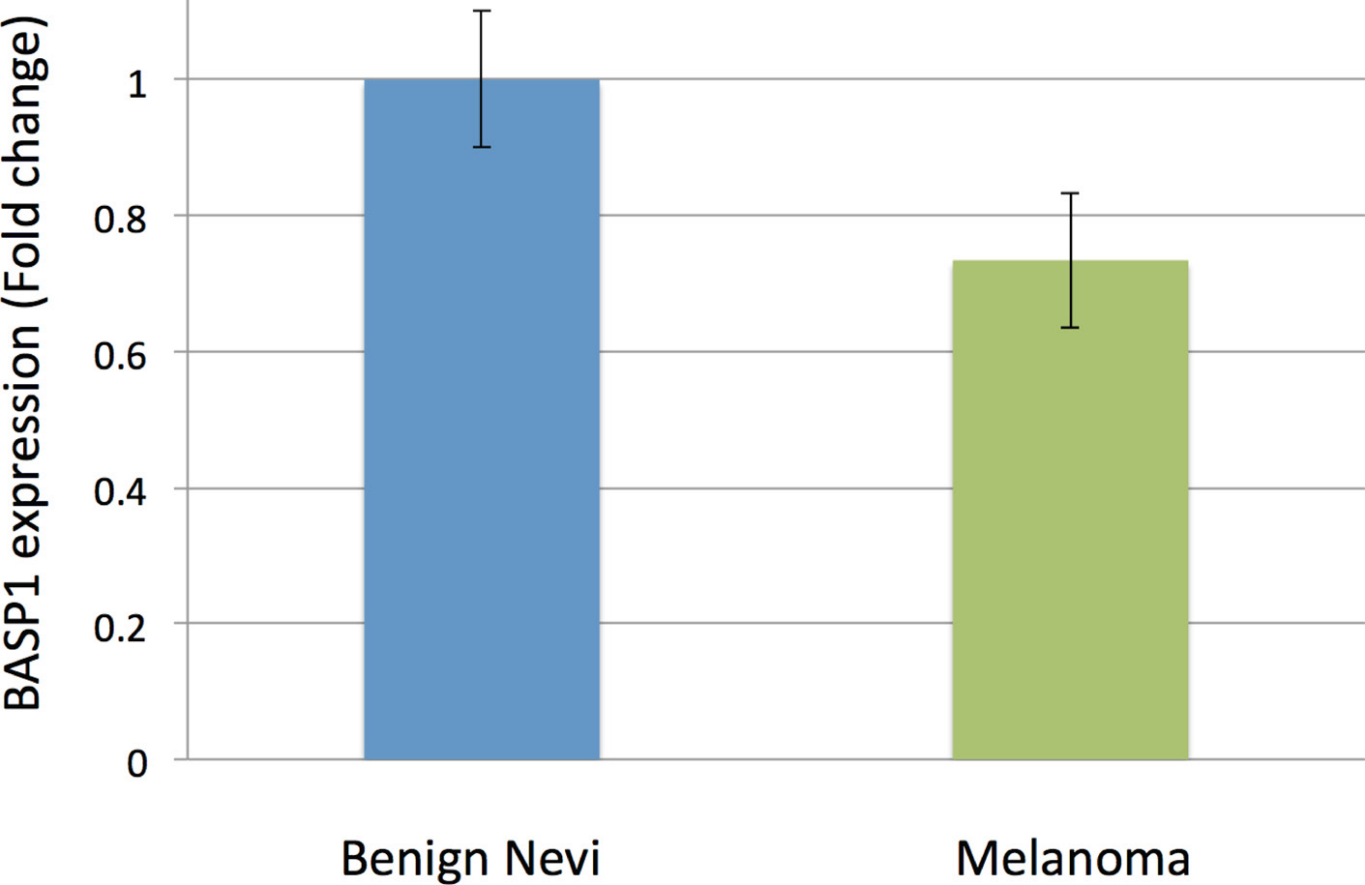
Fold-change in *BASP1* expression in benign nevi (*n* = 18) vs. melanoma samples (*n* = 45) using publicly available data in GEO (GDS1375) Using benign nevi as a reference, *BASP1* is suppressed by about 0.26 fold in melanoma samples (*P* = 0.007, moderated t-statistic). Error bars represent standard error. Data from GEO, GDS1375.

Our study identified one novel SNP not previously associated with melanoma, rs187843643. While rs187843643 did not reach statistical significance in stage 2, likely due to low allele frequency and limited number of stage 2 cases, its 95% confidence interval (for odds ratios) overlapped with corresponding stage 1 confidence intervals. Rs187843643, located at 5p15.1, lies 177 kb downstream of brain abundant membrane attached signal protein 1 (BASP1; *P* = 3.53 × 10^−08^; OR = 1.96, logistic regression) and within a poorly characterized long noncoding RNA, RP11–321E2.4. BASP1 is a protein-coding gene, with several PEST motifs, which are associated with proteins with high turnover. The role of BASP1 protein in cancer has not been well established. One study demonstrated an association between increased BASP1 expression in stage III and stage IV melanoma tumor cells and improved melanoma survival [10]. Consistent with a protective role for BASP1 in melanoma, we found that BASP1 expression was suppressed in melanoma (*N* = 45) as compared with benign nevi (*N* = 18) by 0.26 fold (*P* = 0.007, moderated t-statistic) using publicly available expression data (GEO, GDS1375/GSE3189) (Figure 2) [11, 12]. BASP1 expression has also been shown to be downregulated in hepatocellular carcinoma via epigenetic regulation [13]. This implicates a potential tumor-suppressive role for BASP1 in melanoma. Interestingly, this locus was not identified by the recent meta-analysis by Law et al., potentially due to variability in the imputation panels and QC filters [14].

Telomere homeostasis has been previously associated with melanoma risk. Multiple studies now support the association between longer telomere length and increased melanoma susceptibility, as well with increased nevus count [15, 16]. In addition, telomere-related loci have been associated with risk of melanoma in GWAS including: *ATM, TERT* [17], and more recently, *OBFC1* [14]. TERT, the catalytic subunit of telomerase, plays a critical role in maintaining telomere length and has been shown to support cancer progression through both telomere-dependent and telomere-independent mechanisms [18]. Polymorphisms at the *TERT* locus have been associated with melanoma in multiple candidate studies and rare mutations in *TERT* have been identified in high-incident melanoma families [3]. Our GWAS found an association between the *TERT* marker rs139996880 (5p15.33) and increased melanoma risk at genome-wide significance (*P* = 7.16 × 10^−12^; OR = 1.26) confirming the association of *TERT* with melanoma. Our findings further support the importance of telomere homeostasis in melanoma.

This two-stage GWAS validates the use of consumer self-report data as a platform for discovery of new cancer-related genes, provides confirmation of 20 out of 21 of the previously known melanoma-associated loci, and identifies one novel susceptibility locus (5p15.1; *BASP1*) which confers a 1.96-fold increase in risk of melanoma. Further exploration into the role of the *BASP1* locus in melanoma pathogenesis is warranted.

## MATERIALS AND METHODS

### Stage 1 study design and population

23andMe, Inc. (Mountain View, CA), a personal genetics company, provided free access to aggregated genetic and phenotypic information for stage 1 of the GWAS. 23andMe research participants provided informed consent, in accordance with 23andMe's human subjects protocol (reviewed and approved by Ethical and Independent Review Services, a AAHRPP-accredited IRB). 23andMe gathers genetic information for research by genotyping sample material provided by customers who have consented to research; phenotypic information is collected via online surveys taken by research participants. Inclusion and exclusion criteria are discussed below.

### Stage 1 genome-wide association analysis

Association analysis for stage 1 was performed using logistic regression, assuming an additive model for allelic effects. The analysis was adjusted for age, sex, and population stratification (using the first five principal components), generating the following model:

1) *Melanoma diagnosis ~ age + sex + pc.0 + pc.1 + pc.2 +pc.3 + pc.4 + genotype*. Analyses were restricted to individuals with > 97% European ancestry from the local ancestry analysis to address outlier. Five principal components were extensively evaluated to verify robustness and its use in capturing ancestry structure within Europe. The association test *p value* was computed using a likelihood ratio test. Results for the X chromosome were computed similarly, with male genotypes coded as if they were homozygous diploid for the observed allele. Additional to principal component analysis, test statistics were adjusted for genomic control to correct for residual population stratification persisting after principal component analysis; the genomic control inflation factor was 1.016 (computed from the median *p value* for results that passed quality control). Regions of interest were defined by identifying SNPs with *P* < 10^−5^, then grouping these into intervals separated by gaps of at least 250 kb, and choosing the SNP with the smallest *p value* within each interval.

### Sensitivity and specificity of stage 1 self-reported data

To assess the validity of self-reported phenotypic data in stage 1, 23andMe surveys (pertaining to skin cancer history and pigmentation) were randomly administered to 188 patients seen in Stanford outpatient clinics. The survey answers were then compared to medical records to assess for accuracy with respect to melanoma diagnosis to determine the sensitivity and specificity of the survey responses. *P* values were determined using a Fisher's exact test due to the presence of low frequency events. This sub-study was approved by the Stanford University Institutional Review Board with a waiver of documentation of informed consent.

### Stage 2 study design and population

The study participants were from a hospital-based case-control study of melanoma, for which cases were recruited from among non-Hispanic white patients and controls at MD Anderson between March 1998 and August 2008. Samples and data were available from 931 melanoma patients and 1,026 cancer-free controls (friends of other patients reporting to clinics), which were frequency-matched on age and sex, had completed a comprehensive skin lifestyle questionnaire, and had passed quality control filters for genotyping. This questionnaire was administered by an interviewer to 70% of patients and controls and was self-administered for the remaining 30%. An additional case series comprising 873 individuals presenting for treatment for melanoma at MD Anderson was also included, bringing the total number of melanoma patients to 1,804. The study protocols were approved by the Institutional Review Board at MD Anderson and informed consent was obtained from all participants.

### Stage 2 genome-wide association analysis

Association analysis with risk of melanoma of genotyped SNPs or most likely genotypes from the imputation study was performed using the PLINK –logistic and –covar options. A logistic regression model was built to measure the additive effect of each SNP on susceptibility to melanoma. A likelihood ratio test was performed under the null hypothesis of x2 distribution with one degree of freedom. The first two PCs were included to adjust for population structure [19].

### Meta-analysis

For each SNP, associations in stage 1 and stage 2 were combined in an inverse-variance-weighted meta-analysis using the METAL software [20]. Imputation qualities across batches in 23andMe chips were tested for to pick up variants that have differences in behavior across arrays. Heterogeneity of per-SNP effect sizes in studies contributing to the stage 1, stage 2, and the overall meta-analysis was assessed. All R^2^ and D’ values between individual SNPs were calculated based on the 1000 Genomes Pilot 1 dataset, CEU Population (http://www.broadinstitute.org/mpg/snap/ldsearchpw.php).

### Proportion of familial relative risk

We have used the formula for calculating the proportion of FRR as outlined by the Cancer Oncological Gene-environment Study (COGS) [5]. The odds ratios derived from our meta-analysis of stage 1 and stage 2 are assumed to be relative risks. We estimated the proportion of the familial relative risk (FRR) explained by each SNP (FRR_snp_) as:

$${\text{FRR}}_{\text{snp}}=\left(pr^{2}+q\right)/{\left(pr+q\right)}^{2}$$

Here, the risk allele and alternative allele frequencies are *p* and *q*, respectively, and *r* is the odds ratio for the risk allele. Allele frequencies are derived from the stage 1 population data. Assuming that the loci combine multiplicatively and are not in linkage disequilibrium, the combined effect of all loci is given by:

$${\text{λ}}_{\text{T}}=\prod_{k}{\text{λ}}_{\text{K}}$$

Here, the product is across all loci. The proportion of the familial relative risk attributable to the SNPs, on a log scale, is then given by log(λ_*T*_)/log(λ_*P*_), where λ_*P*_ is the familial relative risk observed in epidemiological studies, assuming an λ_*P*_ for melanoma of 2.19 [14].

### Gene expression analysis

Processed gene expression data for melanoma and nevi (GSE3189) was obtained from the Gene Expression Omnibus (GEO, http://www.ncbi.nlm.nih.gov/geo/). Forty-five melanoma samples and 18 controls (18 benign nevi) were included [12]. Each gene of interest was selected by its proximity to one of the novel risk alleles. For each dataset, Geo2R, which employs a linear-based model for microarray analysis, was utilized to compare gene expression between melanoma and normal skin controls [21]. Significant results were defined as instances of differential gene expression (in melanoma tissue relative to control) reaching *P* < 0.05 the dataset.

## SUPPLEMENTARY MATERIALS FIGURES AND TABLES





## Abbreviations

SNP, single nucleotide polymorphism; GWAS, genome-wide association study.

Acquisition of data: DA Hinds, J Han, Q Wei, KY Sarin, CI Amos, JE Lee.

Analysis and/or interpretation of data: KJ Ransohoff, W Wu, HG Cho, H Chahal, Y Lin, HJ Dai, DA Hinds, J Han, Q Wei, KY Sarin.

Drafting the manuscript: KJ Ransohoff, HG Cho, J Han, KY Sarin.

Revising manuscript critically for important intellectual content: KJ Ransohoff, HG Cho, W Wu, DA Hinds, J Han, KY Sarin.
