# Supplementary material for: Two-stage genome-wide association study identifies a novel susceptibility locus associated with melanoma
Source: Oncotarget. 2017 Feb 9;8(11):17586–92. doi: 10.18632/oncotarget.15230 (PMC5392271; doi:10.18632/oncotarget.15230)
Supplement: Supplementary file 1 [file oncotarget-08-17586-s001.pdf]

Two-stage genome-wide association study identifies a novel susceptibility locus associated with melanoma

Supplementary Materials

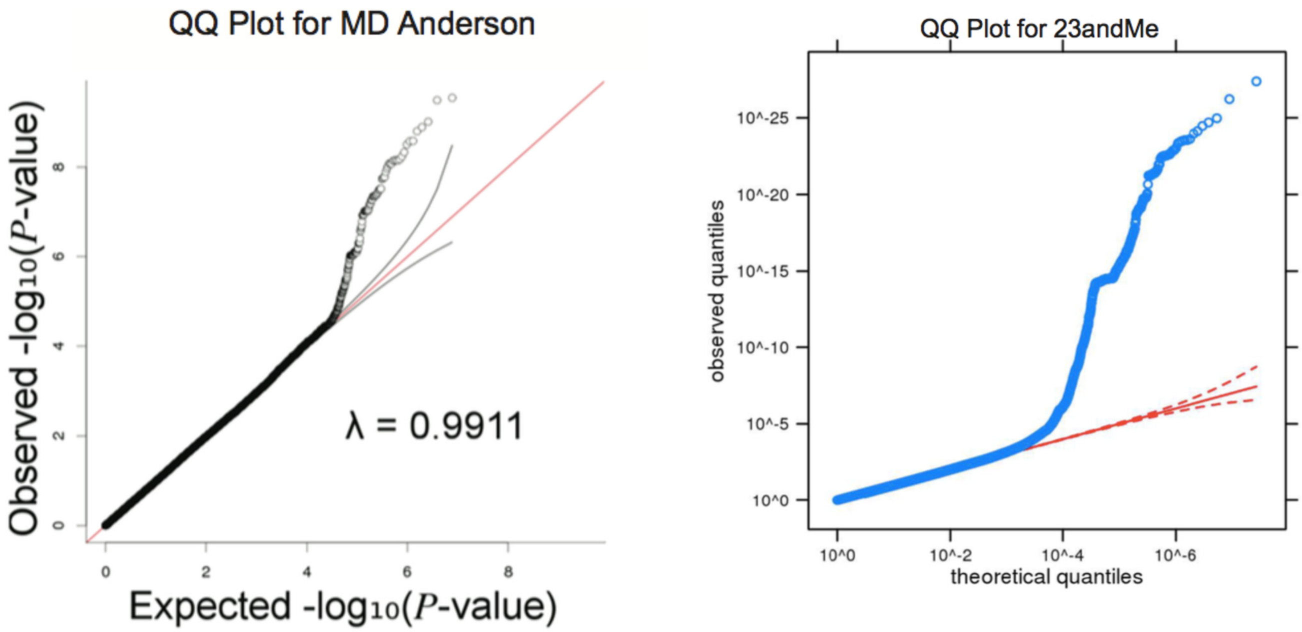

Supplementary Figure 1: QQ plot of observed versus expected quintiles for the melanoma GWAS stage 1 *p* values, plotted on a log scale (top) and for stage 2 (bottom). The null hypothesis states that the expected distribution of p values is uniform. Here, the observed *p* values follow the null distribution for large *p* values ($P > 0.01$) but then diverge for small *p* values. The solid red line has a slope of one and the dashed red lines represent a 95% confidence interval, assuming the test results are independent. The test statistics in the plot have already been adjusted for genomic control.

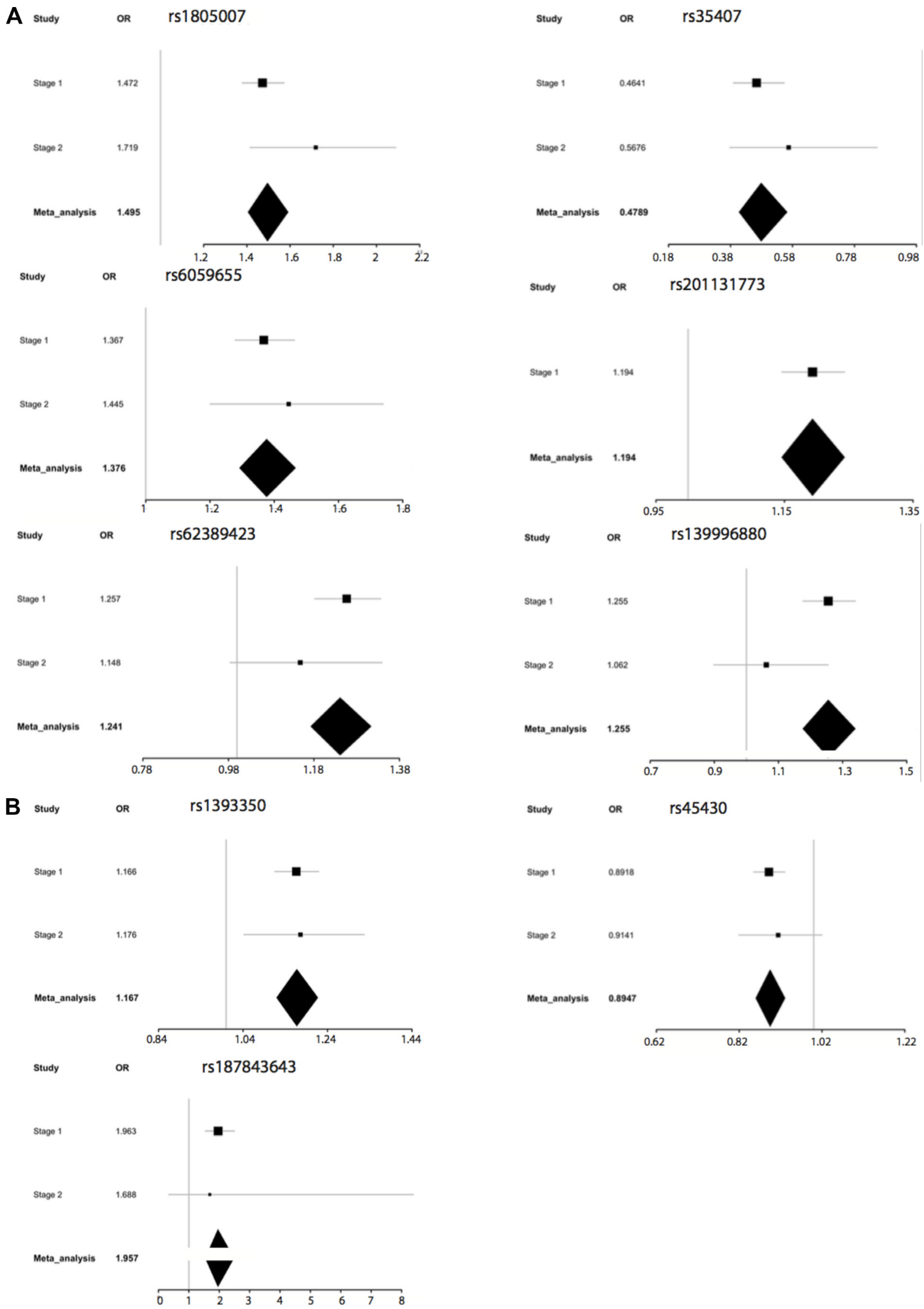

Supplementary Figure 2: Forest plots for each of the nine SNPs reaching genome-wide significance for melanoma. Each plot displays odds ratios from stage 1, stage 2, and overall meta-analysis. For all plots, x-axis displays odds ratio (OR) values and solid vertical lines represent an odds ratio (OR) of 1. The diamond represents the 95% CI for this estimate. Black dots indicate OR from each population and horizontal black lines represent 95% CI.

A

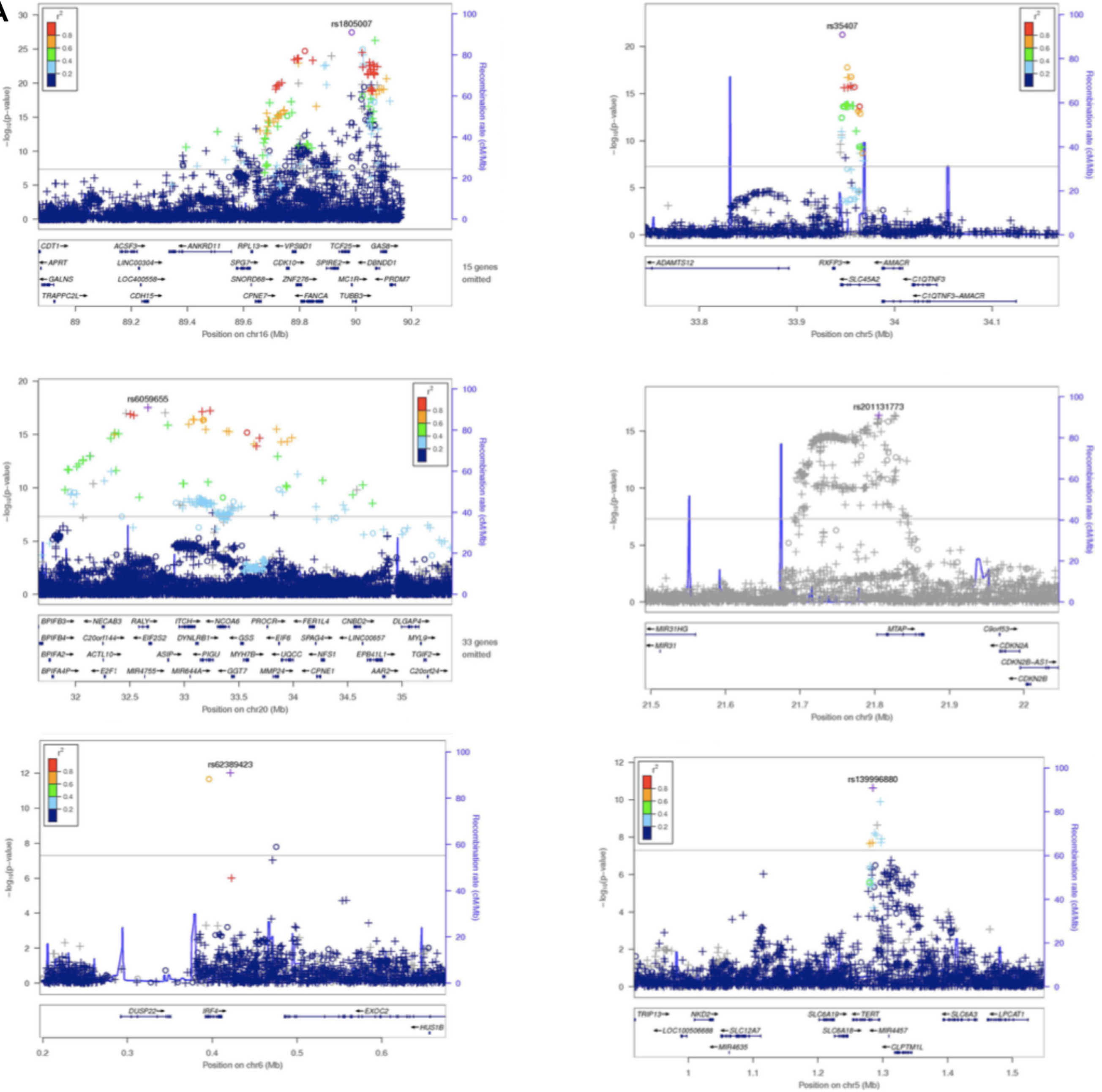
rs1805007
Position on chr16 (Mb)
15 genes omitted
rs35407
Position on chr5 (Mb)
rs6059655
Position on chr20 (Mb)
33 genes omitted
rs201131773
Position on chr9 (Mb)
rs62389423
Position on chr6 (Mb)
rs139996880
Position on chr5 (Mb)
Recombination rate (cM/Mb)

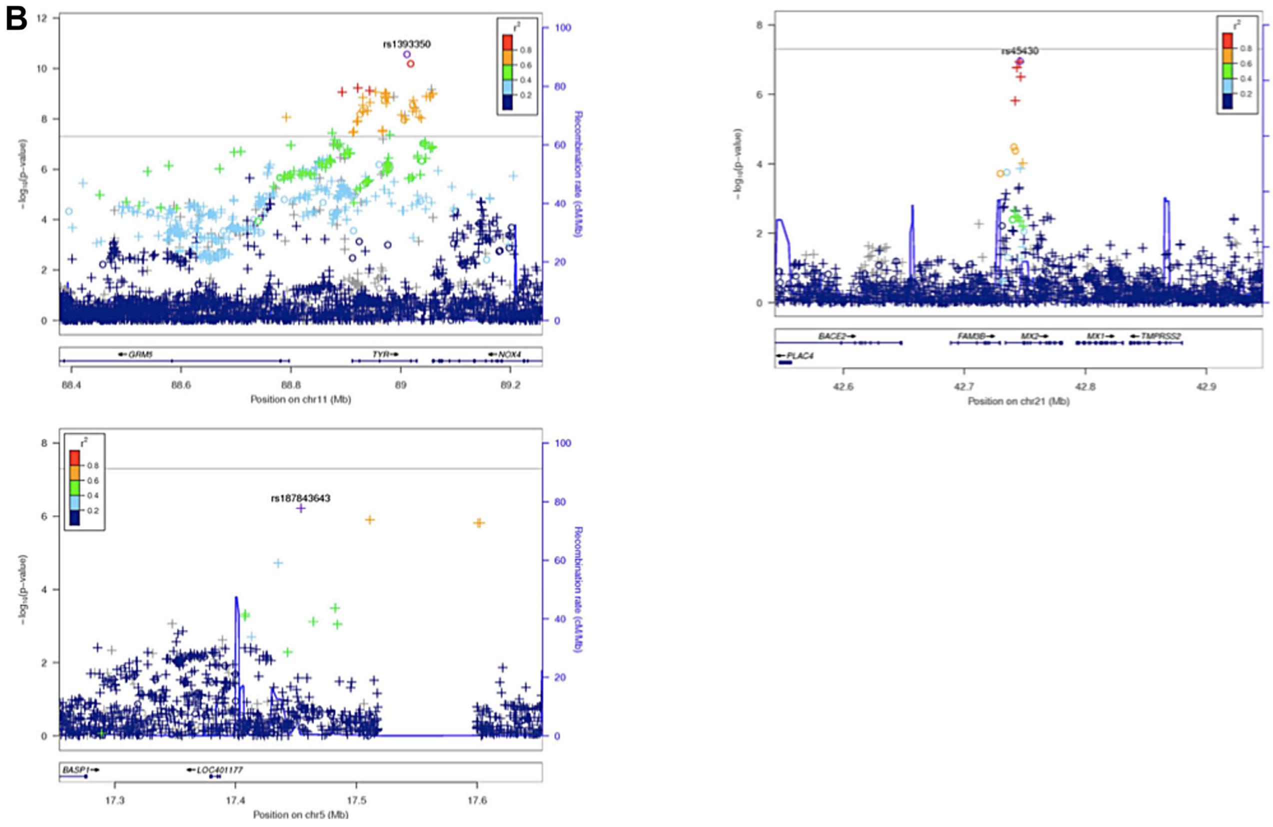

Supplementary Figure 3: Regional association plots for the nine melanoma loci significant at the genome-wide level in stage 1. Each plot is labeled with the rsID for the index SNP corresponding to that locus. Left to right, beginning from top left: 16q24.3 (rs1805007, in between *TCF25-[]-TUBB3*), 5p13.2 (rs35407, *SLC45A2*), 20q11.21 (rs6059655, *RALY-ASIP*), 9p21.3 (rs201131773, *MTAP*), 6p25.3 (rs62389423, in between IRF4 and EXOC2), 5p15.33 (rs139996880, *TERT*), 11q14.3 (rs1393350, *TYR*), 21q22.3 (rs45430, *MX2*), 5p15.1 (rs187843643, *BASP*/Noncoding RNA). Eight out of nine of these are previously known melanoma-associated loci. Each plot displays $-\log_{10}$(P value) versus genomic position based on stage 1 association testing. The color scale indicates strength of linkage disequilibrium (r^2) for nearby SNPs, with respect to the index SNP. To preserve detail, results with $P < 10^{-100}$ are set to 10^{-100}. The "o" and "+" symbols represent genotyped and imputed SNPs, respectively. Recombination rates, in cM/Mb, are also plotted (navy blue lines). These plots were generated via LocusZoom, using LD data from the March 2012 release of 1000 Genomes data.

Stage 1 genotyping and quality control

Samples were genotyped on one of four genotyping platforms. The V1 and V2 platforms were variants of the Illumina HumanHap550+ BeadChip, including about 25,000 custom SNPs selected by 23andMe, with a total of about 560,000 SNPs. The V3 platform was based on the Illumina OmniExpress+ BeadChip, with custom content to improve the overlap with our V2 array, with a total of about 950,000 SNPs. The V4 platform in current use is a fully custom array, including a lower redundancy subset of V2 and V3 SNPs with additional coverage of lower-frequency coding variation, and about 570,000 SNPs. Samples that failed to reach 98.5% call rate were re-analyzed. Individuals whose analyses failed repeatedly were re-contacted by 23andMe customer service to provide additional samples, as is done for all 23andMe customers.

Individuals were only included if they had > 97% European ancestry, as determined through an analysis of local ancestry (1). Briefly, this analysis first partitions phased genomic data into short windows of about 100 SNPs. Within each window, a support vector machine (SVM) is used to classify individual haplotypes into one of 31 reference populations. The SVM classifications are then fed into a hidden Markov model (HMM) that accounts for switch errors and incorrect assignments, and gives probabilities for each reference population in each window. Finally, simulated admixed individuals are used to recalibrate the HMM probabilities so that the reported assignments are consistent with the simulated admixture proportions. The reference population data is derived from public datasets (the Human Genome Diversity Project, HapMap, and 1000 Genomes), as well as 23andMe research participants who have reported having four grandparents from the same country.

A maximal set of unrelated individuals was chosen for each analysis using a segmental identity-by-descent (IBD) estimation algorithm (2). Individuals were defined as related if they shared more than 700 cM IBD, including regions where the two individuals share either one or both genomic segments identical-by-descent. This level of relatedness (roughly 20% of the genome) corresponds approximately to the minimal expected sharing between first cousins in an outbred population.

Research participant genotype data were imputed against the March 2012 "v3" release of 1000 Genomes reference haplotypes (3). Data for each genotyping platform were phased and imputed separately. First, Beagle (4) (version 3.3.1) was used to phase batches of 8,000^{-9},000 individuals across chromosomal segments of no more than 10,000 genotyped SNPs, with overlaps of 200 SNPs. SNPs with Hardy-Weinberg equilibrium $P < 10-20$, call rate < 95%, or with large allele frequency discrepancies compared to European 1000 Genomes reference data were excluded. Frequency discrepancies were identified by computing a 2 × 2 table of allele counts for European 1000 Genomes samples and 2,000 randomly sampled 23andMe research participants with European ancestry, and identifying SNPs with a chi squared $P < 10-15$. Each phased segment was imputed against all-ethnicity 1000 Genomes haplotypes (excluding monomorphic and singleton sites) using Minimac2 (5), using 5 rounds and 200 states for parameter estimation.

For the non-pseudoautosomal region of the X chromosome, males and females were phased together in segments, treating the males as already phased; the pseudoautosomal regions were phased separately. Males and females were then imputed together using minimac, as with the autosomes, treating males as homozygous pseudo-diploids for the non-pseudoautosomal region.

For quality control of genotyped GWAS results, SNPs that were only genotyped on the "V1" platform were flagged due to small sample size, and mitochondrial SNPs or SNPs on the Y chromosome were flagged because many of these are not currently called reliably. Using trio data, SNPs that failed a test for parent-offspring transmission were also flagged; specifically, the child's allele count was regressed against the mean parental allele count, and SNPs with fitted $\beta < 0.6$ and $P < 10-20$ for a test of $\beta < 1$ were flagged. SNPs with a Hardy-Weinberg $P < 10-20$ in Europeans, or a call rate of < 90%, were also flagged. Genotyped SNPs were also tested for genotype date effects, and SNPs with $P < 10-50$ by ANOVA of SNP genotypes against a factor dividing genotyping date into 20 roughly equal-sized buckets were flagged.

For imputed GWAS results, SNPs with average r-squared < 0.5 or minimum r-squared < 0.3 in any imputation batch were flagged, as well as SNPs that had strong evidence of an imputation batch effect. The batch effect test was an F test from an ANOVA of the SNP dosages against a factor representing imputation batch; results with $P < 10-50$ were flagged. Prior to GWAS, the largest subset of the data passing these criteria was identified for each SNP, based on their original genotyping platform — either v2+v3+v4, v3+v4, v3, or v4 only — and association test results were computed for whatever was the largest passing set. As a result, there were no imputed results for SNPs that failed these filters.

When choosing between imputed and genotyped GWAS results, if either the imputed test passed quality control, or a genotyped test was unavailable, the imputed result was reported; otherwise, the genotyped result was reported. For tests using imputed data, imputed dosages were used rather than best-guess genotypes.

Across all results, logistic regression results that did not converge due to complete separation, identified by absolute(effect) > 10 or standard error > 10 on the log odds scale, were flagged. Linear regression results for SNPs with MAF < 0.1% were also flagged, since tests of low frequency variants can be sensitive to violations of the regression assumption of normally distributed residuals. This methodology has been applied in prior GWAS studies (6–8).

Stage 1 phenotype categorization

23andMe identified melanoma cases by using research participants' self-reported answers to online

questionnaires. Subjects who answered "Yes" and/or selected melanoma from a dropdown menu in response to at least one of the following questions were defined as cases: "Have you ever been diagnosed by a doctor with melanoma?" "What type of skin cancer did you have? Please check all that apply." "What type of skin cancer or cancers have you been diagnosed with? Please check all that apply." "Have you ever been diagnosed with melanoma?" "Have you ever been diagnosed or treated for any of the following conditions?" Controls were defined as subjects who answered "No" and did not select melanoma from any relevant dropdown menus. In addition, subjects who answered "No" to at least one of the following questions (and "Yes" to none) were defined as controls: "Have you ever been diagnosed with cancer, including skin cancer or cancerous moles?" "Has a doctor ever told you that you have a type of cancer?" "Have you ever been diagnosed or treated with any of the following conditions?" Among the samples with imputed genotypes, 23andMe had 4,843 melanoma cases and 286,565 controls.

Stage 2 genotyping and quality control

Tissue samples were collected as whole blood, with various DNA extraction methods (including Gentra, Qiagen, and phenol/chloroform). DNA samples for the first-stage genome-wide association study were genotyped using the Illumina Omnil-Quad array and were called using the BeadStudio algorithm, at the John Hopkins University Center for Inherited Disease Research (CIDR).

Mean call rate for all samples was 99.86%. Only 41 failed genotyping with > 10% missing rate across all SNPs, and 11 samples had identity problems that could not be resolved. For this study, the IBD coefficients were estimated using 116,002 autosomal SNPs in PLINK (Purcell et al., 2007). In total, 126 duplicated, related (IBD), or outliers identified by PCA were excluded from the study. Following these exclusions there were 1,952 cases and 1,026 controls. Among 2,978 total cases and controls passing quality control, 138 in situ cases were subsequently removed from the study for indeterminate phenotype. Ten atypical melanocytic proliferation (AMPs) patients were also excluded as not having invasive cancers. Finally, we analyzed data from 1,804 cases and 1,026 controls available for the association study of melanoma susceptibility (Amos et al., 2011). Genome-wide imputation had been applied to case and control samples using MACH program based on 1000 Genome phase I V2 CEU data (2010–11 data freeze, 2012^{-02-14} haplotypes) as a reference panel.

REFERENCES

1. Durand EY, Do CB., Mountain JL, Macpherson JM. http://biorxiv.org/content/early/2014/10/18/010512. Ancestry composition: a novel, efficient pipeline for ancestry deconvolution. (bioRxiv: Cold Spring Harbor Laboratory). 2014.
2. Henn BM, Hon L, Macpherson JM, Eriksson N, Saxonov S, Pe'er I, Mountain JL. Cryptic distant relatives are common in both isolated and cosmopolitan genetic samples. PLoS One. 2012; 7:e34267. doi: 10.1371/journal.pone.0034267.
3. Genomes Project C, Abecasis GR, Altshuler D, Auton A, Brooks LD, Durbin RM, Gibbs RA, Hurles ME, McVean GA. A map of human genome variation from population-scale sequencing. Nature. 2010; 467:1061–73. doi: 10.1038/nature09534.
4. Browning SR, Browning BL. Rapid and accurate haplotype phasing and missing-data inference for whole-genome association studies by use of localized haplotype clustering. Am J Hum Genet. 2007; 81:1084–97. doi: 10.1086/521987.
5. Fuchsberger C, Abecasis GR, Hinds DA. minimac2: faster genotype imputation. Bioinformatics. 2015; 31:782–4. doi: 10.1093/bioinformatics/btu704.
6. Jorgenson E, Makki N, Shen L, Chen DC, Tian C, Eckalbar WL, Hinds D, Ahituv N, Avins A. A genome-wide association study identifies four novel susceptibility loci underlying inguinal hernia. Nat Commun. 2015; 6:10130. doi: 10.1038/ncomms10130.
7. Hu Y, Shmygelska A, Tran D, Eriksson N, Tung JY, Hinds DA. GWAS of 89,283 individuals identifies genetic variants associated with self-reporting of being a morning person. Nat Commun. 2016; 7:10448. doi: 10.1038/ncomms10448.
8. Hinds DA, McMahon G, Kiefer AK, Do CB, Eriksson N, Evans DM, St Pourcain B, Ring SM, Mountain JL, Francke U, Davey-Smith G, Timpson NJ, Tung JY. A genome-wide association meta-analysis of self-reported allergy identifies shared and allergy-specific susceptibility loci. Nat Genet. 2013; 45:907–11. doi: 10.1038/ng.2686.
9. Barrett JH, Iles MM, Harland M, Taylor JC, Aitken JF, Andresen PA, Akslen LA, Armstrong BK, Avril MF, Azizi E, Bakker B, Bergman W, Bianchi-Scarra G, et al. Genome-wide association study identifies three new melanoma susceptibility loci. Nat Genet. 2011; 43: 1108–13. doi: 10.1038/ng.959.
10. Antonopoulou K, Stefanaki I, Lill CM, Chatzinasiou F, Kypreou KP, Karagianni F, Athanasiadis E, Spyrou GM, Ioannidis JP, Bertram L, Evangelou E, Stratigos AJ. Updated field synopsis and systematic meta-analyses of genetic association studies in cutaneous melanoma: the MelGene database. J Invest Dermatol. 2015; 135:1074–9. doi: 10.1038/jid.2014.491.
11. Law MH, Bishop DT, Lee JE, Brossard M, Martin NG, Moses EK, Song F, Barrett JH, Kumar R, Easton DF, Pharoah PD, Swerdlow AJ, Kypreou KP, et al. Genome-wide meta-analysis identifies five new susceptibility loci for cutaneous malignant melanoma. Nat Genet. 2015; 47: 87–95. doi: 10.1038/ng.3373.
12. Bishop DT, Demenais F, Iles MM, Harland M, Taylor JC, Corda E, Randerson-Moor J, Aitken JF, Avril MF, Azizi E, Bakker B, Bianchi-Scarra G, Bressac-de Paillerets B, et al. Genome-wide association study identifies three loci associated with melanoma risk. Nat Genet. 2009; 41:920–5. doi: 10.1038/ng.411.

13. Iles MM, Law MH, Stacey SN, Han J, Fang S, Pfeiffer R, Harland M, Macgregor S, Taylor JC, Aben KK, Akslen LA, Avril MF, Azizi E, et al. A variant in FTO shows association with melanoma risk not due to BMI. Nat Genet. 2013; 45:428–32, 32e1. doi: 10.1038/ng.2571.
14. Song F, Amos CI, Lee JE, Lian CG, Fang S, Liu H, MacGregor S, Iles MM, Law MH, Lindeman NI, Montgomery GW, Duffy DL, Cust AE, et al. Identification of a melanoma susceptibility locus and somatic mutation in TET2. Carcinogenesis. 2014; 35:2097–101. doi: 10.1093/carcin/bgu140.
15. Brown KM, Macgregor S, Montgomery GW, Craig DW, Zhao ZZ, Iyadurai K, Henders AK, Homer N, Campbell MJ, Stark M, Thomas S, Schmid H, Holland EA, et al. Common sequence variants on 20q11.22 confer melanoma susceptibility. Nat Genet. 2008; 40:838–40. doi: 10.1038/ng.163.

Supplementary Table 1: Sensitivity and specificity of self-report data with respect to MM diagnosis

***n* = 186**	**Disease (+)**	**Disease (−)**
Self-report (+)	15	2
Self-report (−)	0	169

Sensitivity = 100%.

Specificity = 98.8%.

Within table, from left to right, are counts for true positives, false positives, false negatives, and true negatives. Data from 186 randomly selected.

Supplementary Table 2: Replication of 21 previously reported significant MM-associated loci

								**Stage 1**		**Stage 2**		**Meta-analysis**		**Prior Studies**
**SNP**	**Ch.**	**Gene**	**Min**	**MAF**	**r2**	***P***	***OR***	***P***	***OR***	***P***	***OR***	***P***	***OR***	**Ref**
rs7412746	1	ARNT	T	0.47	0.99	2.46×10^{-4}	1.08	1.92×10^{-3}	1.19	6.10×10^{-6}	1.09	9.00×10^{-11}	1.15	(9)
rs13016963	2	CASP8	A	0.38	1.00	6.93×10^{-3}	0.94	3.51×10^{-2}	1.13	1.08×10^{-3}	1.07	9.00×10^{-10}	1.14	(10), (11)
rs6750047	2	RMDN2 (CYP1B1)	A	0.28	0.99	2.48×10^{-1}	1.02	8.89×10^{-2}	1.10	9.22×10^{-2}	1.03	7.00×10^{-9}	1.10	(12)
rs16891982	5	SLC45A2	C	0.04	1.00	9.02×10^{-19}	0.48	5.33×10^{-3}	0.58	1.08×10^{-16}	0.50	1.47×10^{-23}	0.42	(11)
rs6914598	6	CDKAL1	C	0.31	0.96	2.22×10^{-2}	1.05	2.52×10^{-1}	1.07	1.10×10^{-2}	1.06	3.50×10^{-8}	1.10	(12)
rs1636744	7	AGR3	T	0.41	1.00	5.59×10^{-2}	1.04	4.99×10^{-2}	1.12	1.31×10^{-2}	1.05	7.10×10^{-9}	1.10	(12)
rs10739221	9	TMEM38B (RAD23B, TAL2)	T	0.24	0.96	7.39×10^{-5}	1.10	2.02×10^{-3}	1.22	1.46×10^{-6}	1.12	7.10×10^{-11}	1.13	(12)
rs7023329	9	MTAP (CDKN2A)	G	0.50	1.00	2.29×10^{-13}	0.86	1.49×10^{-5}	0.79	5.38×10^{-17}	0.85	7.00×10^{-9}	0.86	(10)
rs2995264	10	OBFC1	G	0.10	0.96	2.75×10^{-5}	1.16	2.59×10^{-2}	1.23	1.80×10^{-6}	1.17	2.20×10^{-9}	1.17	(12)
rs1393350	11	TYR	A	0.27	1.00	1.90×10^{-11}	1.17	8.47×10^{-3}	1.18	3.65×10^{-13}	1.17	2.00×10^{-14}	1.29	(13), (10)
rs498136	11	CCND1	A	0.36	0.99	$8.98E \times 10^{-5}$	1.09	1.45×10^{-1}	1.09	2.77×10^{-5}	1.09	1.50×10^{-12}	1.12	(12)
rs1801516	11	ATM	A	0.14	1.00	7.42×10^{-2}	0.95	7.57×10^{-3}	0.81	9.45×10^{-3}	0.93	3.00×10^{-9}	0.88	(10)
rs4778138	15	OCA2	G	0.15	1.00	5.68×10^{-4}	0.90	6.52×10^{-4}	0.75	1.12×10^{-5}	0.88	2.20×10^{-11}	0.84	(12)
rs258322	16	MC1R	A	0.09	1.00	3.87×10^{-16}	1.31	5.32×10^{-6}	1.52	4.48×10^{-21}	1.33	3.00×10^{-27}	1.67	(13), (10)
rs4785763	16	AFG3L1P	A	0.33	1.00	3.47×10^{-18}	1.21	1.64×10^{-6}	1.32	5.21×10^{-23}	1.22	6.00×10^{-22}	1.32	(13) (11)
rs16953002	16	FTO	A	0.17	1.00	7.54×10^{-1}	1.01	5.61×10^{-2}	1.15	3.41×10^{-1}	1.03	3.60×10^{-12}	1.16	(11,14)
rs258322	16	CDK10	A	0.09	1.00	3.87×10^{-16}	1.31	5.32×10^{-6}	1.52	4.48×10^{-21}	1.33	2.00×10^{-9}	1.67	(13), (15)
rs1885120	20	MYH7B	C	0.07	0.98	4.02×10^{-16}	1.34	1.77×10^{-4}	1.43	3.71×10^{-20}	1.35	1.60×10^{-18}	1.55	(11)
rs910873	20	PIGU	A	0.08	1.00	2.61×10^{-17}	1.35	1.66×10^{-4}	1.43	1.85×10^{-21}	1.36	9.90×10^{-16}	1.75	(16)
rs45430	21	MX2	C	0.40	1.00	8.80×10^{-8}	0.89	1.08×10^{-1}	0.91	2.89×10^{-8}	0.90	3.00×10^{-9}	0.88	(10)
rs2284063	22	PLA2G6	G	0.36	0.99	1.21×10^{-2}	0.95	3.83×10^{-3}	0.84	8.15×10^{-4}	0.93	2.00×10^{-9}	0.83	(13)

21 loci previously confirmed as associated with MM via prior GWAS ($P < 5 \times 10^{-8}$) are listed, all of which independently reached nominal significance ($P < 0.05$) in this study. Additionally, we report chromosome, nearest genes, minor allele, minor allele frequency (MAF) as calculated from stage 1 controls, average imputation r^2 (a measure of imputation quality) for stage 1, and odds ratio (OR) with *P* value for each stage, calculated with respect to the minor allele. The right-most 3 columns list *P* value and OR from prior publications for each locus, relative to minor allele, along with corresponding reference. Statistics for effect heterogeneity (P_{het} and I^2) are included in Supplementary Table 4.
MAF = minor allele frequency in stage 1 controls.

Supplementary Table 3: Imputation and effect heterogeneity statistics for 9 genome-wide significant SNPs

			Stage 1			Stage 2			Meta-analysis	
**SNP**	**Gene**	**Maj/Min**	**MAF**	**avg r^2**	**min r^2**	**MAF**	**imputation r^2**	**Genotyped**	**P_{het}**	**I^2**
rs1805007	*MC1R*	C/T	0.07	1.00	1.00	0.07	0.74	Imputed	0.14	54.2
rs35407	*SLC45A2*	G/A	0.04	0.98	0.88	0.02	_	Genotyped	0.37	0
rs6059655	*RALY (ASIP)*	G/A	0.07	0.99	0.98	0.09	0.98	Imputed	0.58	0
rs201131773	*MTAP*	I/D	0.48	0.99	0.98	-	-	-	1.00	0
rs62389423	*IRF4-[]--EXOC2*	G/A	0.14	0.78	0.76	0.14	0.81	Imputed	0.28	12.7
rs139996880	*TERT*	G/A	0.16	0.65	0.55	0.12	0.30	Imputed	1.00	0
rs1393350	*TYR*	G/A	0.26	1.00	1.00	0.28	_	Genotyped	0.90	0
rs45430	*MX2*	T/C	0.40	1.00	0.99	0.39	_	Genotyped	0.68	0
rs187843643	*BASP1---[]***	C/T	0.01	0.74	0.61	0.00	0.41	Imputed	0.86	0

SNPs that met genome-wide significance ($P < 5 \times 10–8$) in stage 1 and/or overall meta-analysis are listed. Additionally, we report genetic context, minor alleles, stage 1 minor allele frequency (MAF), stage 1 average imputation r^2 (avg r^2), stage 1 minimum imputation r^2, stage 2 MAF, stage 2 average imputation r^2 and overall, and P value (P_{het}) and I^2 for effect heterogeneity pertaining to meta-analysis of combined stage 1-stage 2 data.

Supplementary Table 4: Imputation and effect heterogeneity statistics for previously reported SNPs.

See_Supplementary_Table 4
