# Supplementary material for: Two-stage genome-wide association study identifies a novel susceptibility locus associated with melanoma
Source: Oncotarget. 2017 Feb 9;8(11):17586–92. doi: 10.18632/oncotarget.15230 (PMC5392271; doi:10.18632/oncotarget.15230)
Supplement: Supplementary file 2 [file oncotarget-08-17586-s002.docx]

Stage 1

Stage 2

Meta-analysis

**Supplementary Table S4. Imputation and effect heterogeneity statistics for previously reported SNPs**

| SNP | Gene | Min | MAF | avg r^2^ | min r^2^ | MAF | Imputation r^2^ |  | *P_het_* | *I*^2^ |
| --- | --- | --- | --- | --- | --- | --- | --- | --- | --- | --- |
|  |  |  |  |  |  |  |  |  |  |  |
| rs7412746 | *ARNT* | T | 0.47 | 0.99 | 0.97 | 0.47 | _ | Genotyped | 0.11 | 61 |
| rs13016963 | *CASP8* | A | 0.47 | 1.00 | 1.00 | 0.36 | 1.00 | Imputed | 0.30 | 6.5 |
| rs6750047 | *RMDN2 (CYP1B1)* | A | 0.28 | 0.99 | 0.99 | 0.49 | 0.96 | Imputed | 0.24 | 28.8 |
| rs16891982 | *SLC45A2* | C | 0.05 | 1.00 | 0.99 | 0.03 | 0.91 | Imputed | 0.38 | 0 |
| rs6914598 | *CDKAL1* | C | 0.31 | 0.96 | 0.93 | 0.31 | 0.96 | Imputed | 0.80 | 0 |
| rs1636744 | *AGR3* | A | 0.40 | 1.00 | 0.97 | 0.40 | _ | Genotyped | 0.24 | 28.2 |
| rs10739221 | *TMEM38B (RAD23B,TAL2)* | T | 0.24 | 0.96 | 0.95 | 0.22 | 0.94 | Imputed | 0.14 | 55.2 |
| rs7023329 | *MTAP(CDKN2A)* | G | 0.50 | 1.00 | 1.00 | 0.49 | _ | Genotyped | 0.14 | 53.8 |
| rs2995264 | *OBFC1* | G | 0.09 | 0.96 | 0.93 | 0.09 | 0.99 | Imputed | 0.55 | 0 |
| rs1393350 | *TYR* | A | 0.01 | 1.00 | 1.00 | 0.28 | _ | Genotyped | 0.90 | 0 |
| rs1126809 | *TYR* | A | 0.26 | 0.99 | 0.98 | 0.29 | 0.96 | Imputed | 0.37 | 0 |
| rs1847134 | *TYR* | A | 0.14 | 1.00 | 1.00 | 0.32 | _ | Genotyped | 0.43 | 0 |
| rs498136 | *CCND1* | A | 0.36 | 0.99 | 0.99 | 0.35 | 0.97 | Imputed | 0.99 | 0 |
| rs1801516 | *ATM* | A | 0.28 | 1.00 | 1.00 | 0.15 | _ | Genotyped | 0.06 | 71.8 |
| rs17655 | *ATM* | C | 0.31 | 1.00 | 1.00 | 0.21 | _ | Genotyped | 0.66 | 0 |
| rs4778138 | *OCA2* | A | 0.15 | 1.00 | 0.99 | 0.14 | _ | Genotyped | 0.05 | 74.4 |
| rs258322 | *MC1R* | A | 0.09 | 1.00 | 1.00 | 0.09 | _ | Genotyped | 0.12 | 58.8 |
| rs4785763 | *MC1R* | A | 0.09 | 1.00 | 1.00 | 0.33 | _ | Genotyped | 0.15 | 52.4 |
| rs16953002 | *FTO* | A | 0.09 | 1.00 | 1.00 | 0.16 | _ | Genotyped | 0.09 | 64.6 |
| rs258322 | *CDK10* | A | 0.09 | 1.00 | 1.00 | 0.09 | _ | Genotyped | 0.12 | 58.8 |
| rs1885120 | *MYH7B* | C | 0.09 | 0.98 | 0.84 | 0.09 | _ | Genotyped | 0.53 | 0 |
| rs910873 | *PIGU* | A | 0.07 | 1.00 | 0.94 | 0.09 | _ | Genotyped | 0.57 | 0 |
| rs45430 | *MX2* | C | 0.07 | 1.00 | 0.99 | 0.39 | _ | Genotyped | 0.68 | 0 |
| rs2284063 | *PLA2G6* | G | 0.4 | 0.99 | 0.99 | 0.37 | 1.00 | Imputed | 0.07 | 70.7 |

These SNPs are all previously reported in prior MM publications (21 SNPs). We report genetic context, major and minor alleles, stage 1 minor allele frequency (MAF), stage 1 average imputation r^2^ (avg r^2^), stage 1 minimum imputation r^2^, stage 2 MAF, stage 2 average imputation r^2^ and overall, and *P* value (*P_het_*) and *I^2^* for effect heterogeneity pertaining to meta-analysis of combined stage 1-stage 2 data.
